# Multiparametric Quantitative Ultrasound for Hepatic Steatosis: Comparison with CAP and Robustness Across Breathing States

**DOI:** 10.3390/diagnostics15243119

**Published:** 2025-12-08

**Authors:** Alexandru Popa, Ioan Sporea, Roxana Șirli, Renata Bende, Alina Popescu, Mirela Dănilă, Camelia Nica, Călin Burciu, Bogdan Miutescu, Andreea Borlea, Dana Stoian, Felix Maralescu, Eyad Gadour, Felix Bende

**Affiliations:** 1 Department of Internal Medicine II, Division of Gastroenterology and Hepatology, “Victor Babeș” University of Medicine and Pharmacy, 300041 Timișoara, Romania; popa.alexandru@umft.ro (A.P.); miutescu.bogdan@umft.ro (B.M.); bende.felix@umft.ro (F.B.); 2 Center for Advanced Research in Gastroenterology and Hepatology, “Victor Babeș” University of Medicine and Pharmacy, 300041 Timișoara, Romania; 3 Department of Gastroenterology, Faculty of Medicine, Pharmacy and Dental Medicine, “Vasile Goldiș” West University of Arad, 310414 Arad, Romania; 4 Department of Internal Medicine II, Division of Endocrinology, “Victor Babeș” University of Medicine and Pharmacy, E. Murgu Square, Nr. 2, 300041 Timișoara, Romania; 5 Department of Internal Medicine II, Discipline of Nephrology, “Victor Babeș” University of Medicine and Pharmacy, 300041 Timișoara, Romania; mihai.maralescu@umft.ro; 6 Multi-Organ Transplant Centre of Excellence, Liver Transplantation Unit, King Fahad Specialist Hospital, Dammam 32253, Saudi Arabia

**Keywords:** hepatic steatosis, quantitative ultrasound, attenuation coefficient, ultrasound-derived fat fraction

## Abstract

**Background:** Practical, quantitative ultrasound-based tools for measuring hepatic steatosis are needed in everyday MASLD care. We evaluated a new multiparametric quantitative ultrasound (QUS) platform that integrates ultrasound-guided fat fraction (UGFF), attenuation coefficient (AC), backscatter coefficient (BSC), and signal-to-noise ratio (SNR), using Controlled Attenuation Parameter (CAP) as the reference and examining the effect of breathing. **Methods:** In a prospective single-center study, adult patients underwent same-day liver QUS and FibroScan. QUS measurements were performed during breath-hold and during normal breathing. Regions of interest were placed in right-lobe parenchyma 2 cm below the capsule, avoiding vessels. Primary outcomes were correlation with CAP and ROC performance at CAP cutoffs for S1 (≥230 dB/m), S2 (≥275 dB/m), and S3 (≥300 dB/m). **Results:** QUS was feasible in almost all examinations. UGFF, BSC, and SNR were consistent across breathing conditions, while AC was slightly higher during normal breathing. UGFF showed strong correlation with CAP and high accuracy for detecting steatosis. Across grades, AUCs were around 0.89–0.91, with cutoffs (UGFF ≈ 4% for ≥S1 and ≈11% for ≥S3). **Conclusions:** Multiparametric QUS provides reliable liver fat quantification that aligns closely with CAP and remains robust in practice whether patients hold their breath or breathe normally. These findings support UGFF as a practical, reliable point-of-care alternative for liver fat quantification that can be embedded in routine ultrasound in real time. Validation against MRI-PDFF or histology and multicenter studies will further define cutoffs and generalizability.

## 1. Introduction

Fatty liver disease is currently the most common liver condition encountered in hepatology practice. In previous decades, the main focus of hepatology research and clinical management centered on chronic viral hepatitis (HBV, HCV, HDV); today, however, the therapeutic landscape has shifted dramatically [1,2]. The introduction of highly effective direct-acting antivirals capable of eradicating HCV infection within 8–12 weeks and the widespread availability of nucleos(t)ide analogs that achieve durable HBV viral suppression, such as entecavir and tenofovir, have reduced the global burden of viral hepatitis. Consequently, research emphasis has moved toward other liver diseases in which early detection, accurate staging, and effective management are still major challenges. Although alcoholic liver disease (ALD) remains highly prevalent, it continues to remain relatively under-resourced compared with its burden. In contrast, fatty liver disease—initially referred to as non-alcoholic fatty liver disease (NAFLD)—has rapidly emerged as the most common chronic liver disorder worldwide, now representing a key topic in hepatology and internal medicine. Recent epidemiological data confirm this paradigm shift. Younossi et al. reported that approximately 30% of the global population is affected by fatty liver disease, with prevalence rates ranging from 25% in Europe to 44% in South America and 34% in South Asia. These numbers have increased compared with earlier reports estimating 30.4% and 23.7% for South America and Europe, respectively [1,2]. The burden is even higher in high-risk populations: among patients with type 2 diabetes mellitus (T2DM), the global prevalence of fatty liver disease reaches 55.5%, with Europe showing the highest rate—68% [3]. In a meta-analysis of more than 1.8 million T2DM patients, Cho EEL et al. reported that 65.0% had fatty liver, 31.5% had steatohepatitis (MASH), 35.5% had clinically significant fibrosis (F2–F4), and 14.9% already had advanced fibrosis (F3–F4) [4].

For years, NAFLD was the standard term describing fatty liver not due to significant alcohol consumption. However, growing recognition of metabolic dysfunction as the core pathogenic driver led to the proposal of MAFLD (metabolically associated fatty liver disease) [5]. In 2023, an international multisociety consensus recommended the terminology MASLD (metabolic dysfunction-associated steatotic liver disease) to harmonize nomenclature and emphasize the metabolic substrate of the disease [6].

The risk factors for MASLD—obesity, T2DM, dyslipidemia, and metabolic syndrome—are well established. Given its massive prevalence, the key challenge for clinicians and health systems is how to identify and stage steatosis efficiently, cost-effectively, and non-invasively across large populations. Ultrasound is widely accepted as the first-line imaging modality for liver evaluation because it is inexpensive, radiation-free, and readily available. Conventional B-mode ultrasound detects fatty infiltration by increased hepatic echogenicity, but this assessment is qualitative and operator-dependent, with limited sensitivity for mild steatosis (≈50–60% when fat is below 5%) [7]. Computed tomography (CT) and magnetic resonance imaging (MRI) can quantify fat more precisely, but CT entails ionizing radiation and MRI, while highly accurate—especially MRI-proton-density-fat-fraction (MRI-PDFF)—is expensive and less accessible, limiting its suitability for large-scale screening. Thus, new ultrasound-based quantitative approaches have been developed to bridge this diagnostic gap.

Accurate quantification of liver fat content is essential for risk stratification and for monitoring response to therapy. The Controlled Attenuation Parameter (CAP), implemented in FibroScan^®^ (Echosens), is currently the most widely adopted quantitative tool. CAP measures ultrasound attenuation in the 2–5 MHz range, during transient elastography, and has been validated against biopsy and MRI-PDFF [8,9,10,11]. Its advantages include standardization and high reproducibility; however, CAP is “blind,” lacking real-time imaging guidance, and its values may be affected by factors such as subcutaneous fat, probe selection (M vs. XL), and heterogeneous parenchymal pathology [11].

Recent technological advances now allow quantitative ultrasound (QUS) parameters—such as the attenuation coefficient and backscatter coefficient—to be measured directly on ultrasound systems under B-mode guidance [12,13]. These metrics correlate strongly with hepatic fat fraction because fatty infiltration increases both attenuation and backscatter of the ultrasound beam. Additional parameters, such as signal-to-noise ratio or acoustic structure quantification indices, provide further quality or texture information [14].

By combining these complementary acoustic biomarkers, newer ultrasound platforms have enabled sophisticated tissue characterization using both physical and statistical modeling. QUS parameters, such as the attenuation coefficient (AC), integrated backscatter coefficient (BSC), and signal-to-noise ratio (SNR), have emerged as quantifiable imaging biomarkers for hepatic steatosis. The ultrasound-guided fat fraction (UGFF) algorithm integrates these multiparametric acoustic features to enhance the accuracy and reproducibility of liver fat quantification [15]. Early studies have demonstrated strong correlations between various ultrasound fat fraction techniques and MRI-PDFF or CAP, with AUROC values typically between 0.88 and 0.95 for detecting ≥S1 steatosis.

Given the epidemiological importance of MASLD and the limitations of existing tools, the development of reliable, multiparametric ultrasound-based quantification methods is of high clinical relevance. The present prospective study aimed to evaluate the feasibility and diagnostic performance of a new multiparametric QUS technique—integrating UGFF (%), AC (dB/m), BSC (dB), and SNR—for hepatic steatosis assessment.

Measurements were performed on an advanced ultrasound platform and compared with the established CAP values obtained from FibroScan^®^ (Echosens, Paris, France).

Specifically, the objectives of this paper were to assess the reliability of QUS measurements under different breathing conditions (apnea vs. free breathing), to analyze the correlations between each QUS parameter and CAP, and to evaluate the diagnostic accuracy of all parameters (UGFF, AC, BSC, SNR) for detecting and grading hepatic steatosis (≥S1, ≥S2, and S3), with the overarching aim of determining whether multiparametric QUS can function as a practical, imaging-guided alternative to CAP and provide a comprehensive, point-of-care solution for non-invasive liver fat quantification.

## 2. Materials and Methods

### 2.1. Study Design and Setting

A single-center, prospective observational study was conducted in the Department of Gastroenterology and Hepatology, “Victor Babeș” University of Medicine and Pharmacy, Timișoara, Romania. Subject enrollment took place between May and August 2025. The methodology focused on assessing a multiparametric quantitative ultrasound (QUS) platform—which incorporates ultrasound fat fraction (UGFF,%), attenuation coefficient (AC, dB/m), backscatter coefficient (BSC, dB), and signal-to-noise ratio (SNR)—in comparison with the Controlled Attenuation Parameter (CAP, dB/m) as measured by FibroScan^®^ Compact 530, Echosens, Paris, France. The study protocol was approved by the Institutional Ethics Committee on 28 April 2025 (protocol code [SCJUPBT-32/2025]) and conducted in accordance with the Declaration of Helsinki. Written informed consent was obtained from all participants.

### 2.2. Study Population

A total of 196 consecutive adults, including both patients and volunteers, took part in same-day liver ultrasound, QUS (UGFF, AC, BSC, SNR), and CAP assessments during the study period. The cohort was intentionally assembled to cover the full spectrum of hepatic fat burden (from no steatosis to severe steatosis). Baseline demographic and laboratory data (for patients) were collected prospectively and are summarized in Table 1.

### 2.3. Eligibility Criteria

Inclusion criteria: age ≥ 18 years; written informed consent; completion of both QUS (UGFF, AC, BSC, SNR) and CAP during the same visit.

Exclusion criteria: absence of consent; pregnancy; active implantable cardiac device;ascites; known or suspected decompensated cirrhosis (history or imaging evidence of portal hypertension/decompensation); active chronic viral hepatitis (HBV or HCV), autoimmune hepatitis, primary biliary cholangitis, primary sclerosing cholangitis; focal liver lesions suspicious for primary or secondary malignancy on B-mode ultrasound; recent (<6 months) major hepatic surgery, locoregional hepatic therapy (e.g., TACE, RFA), or bariatric surgery; marked cytolysis (AST or ALT > 5× upper limit of normal); inability to obtain ≥10 valid CAP measurements or QUS acquisitions of acceptable quality.

### 2.4. Patient Preparation and Examination Workflow

All liver examinations adhered to EFSUMB and WFUMB recommendations [13,16]. Participants fasted for ≥4 h and rested for ≥10 min before imaging. QUS and CAP were obtained on the same day, under a standard operating procedure to limit temporal variability. Operators were blinded to the results of the other modality during acquisition and initial data entry. Two experienced examiners (each with >5 years in hepatic ultrasound and elastography) performed all measurements after joint protocol training and alternated between QUS and CAP to reduce operator effects.

### 2.5. QUS Protocol (UGFF, AC, BSC, SNR)

QUS was acquired on a LOGIQ™ E10 system (GE HealthCare, Chicago, IL, USA) with a C1–6 convex transducer and a research/prototype multiparametric module. Patients were examined in the supine position with the right arm maximally abducted; measurements were obtained via a right intercostal approach targeting segments V/VIII. Machine presets (output power, frequency, dynamic range, overall gain, and TGC) were kept constant; liberal gel and minimal probe pressure were used to ensure stable coupling and to limit compression artifacts.

#### 2.5.1. Workflow

(i) A standard B-mode survey was performed and a relatively homogeneous liver section was identified, with minimal interference from large vessels, rib shadowing, or other anatomical structures.

(ii) UGFF mode was activated. Once an appropriate B-mode image of the parenchyma was obtained, UGFF data acquisition was started; because the algorithm operates on B-mode signals, a few seconds of scanning were sufficient to collect the necessary frames.

(iii) Freeze was pressed to measure UGFF from the cine loop. The ROI was initially placed centrally and then adjusted laterally using the trackball to avoid inclusion of large vessels or artifacts. In line with the vendor-described UGFF workflow, the platform includes an automatic measurement adjustment algorithm intended to yield results consistent with the WFUMB-recommended ROI placement (~2 cm below the capsule) without requiring manual measurement of the liver-surface distance; this approach reduces dependence of the final estimate on exact ROI depth positioning.

(iv) Once the ROI was optimized, pressing Set immediately displayed the calculated UGFF (%); the next cine frame was automatically selected and the procedure was repeated.

In this study, to assess repeatability and the effect of respiration, 20 consecutive measurements were obtained in the same segment—10 during a brief breath-hold (apnea) and 10 during quiet breathing. For each acquisition the console displayed UGFF (%), AC (dB/m), BSC (dB), and SNR.

#### 2.5.2. Ultrasound-Guided Fat Fraction (UGFF)

Ultrasound-guided fat fraction (UGFF) is reported on a 0–100% scale. It is a proprietary multivariate estimator that integrates complementary QUS features—attenuation (AC, dB/m), compensated backscatter (BSC), and texture homogeneity (SNR, computed as the mean-to-standard-deviation ratio of the linear envelope from tissue-harmonic data). These features are extracted from fundamental and tissue-harmonic B-mode frames acquired under fixed transmit/receive settings and combined on-system to yield a single fat-fraction output (UGFF, %). UGFF reflects the relative contribution of lipid-related acoustic changes in the parenchyma and is reported as the median across valid acquisitions. In this study, UGFF was summarized per condition (apnea and quiet breathing) as the median of 10 valid acquisitions.

#### 2.5.3. Attenuation Coefficient (AC, UGAP)

AC quantifies the frequency-dependent loss of acoustic energy with depth. As the amount of fat droplets in the liver increases, the absorption of the ultrasound propagation increases, resulting in deep attenuation. Values are computed in the selected ROI using a spectral/log-spectral method with depth compensation and spatial averaging, yielding the slope of attenuation versus frequency. Higher AC values indicate increased attenuation, as typically seen with hepatic steatosis. The attenuation coefficient (AC) can be expressed as dB/m or dB/cm/MHz; for consistency and direct comparability with CAP, we report AC exclusively in dB/m throughout.

#### 2.5.4. Backscatter Coefficient (BSC)

BSC is a new QUS parameter. It quantifies the intensity of echo signals (brightness of B-mode images). As the amount of fat droplets in the liver increases, the backscatter also increases, resulting in higher echo signal intensity.

#### 2.5.5. Signal-to-Noise Ratio (SNR)

**Figure 1:**
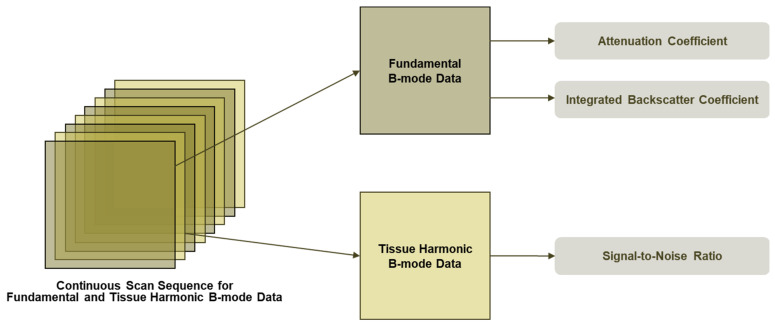
Continuous scan sequence for fundamental and tissue-harmonic B-mode base data. Fundamental frames are used to estimate the attenuation coefficient (AC) and integrated backscatter coefficient (BSC), while tissue-harmonic frames are used to compute the signal-to-noise ratio (SNR). All frames are acquired in a single interleaved sequence with fixed transmit/receive settings, enabling quantitative outputs that are subsequently integrated on-system to yield the ultrasound-guided fat fraction (UGFF).

**Figure 2:**
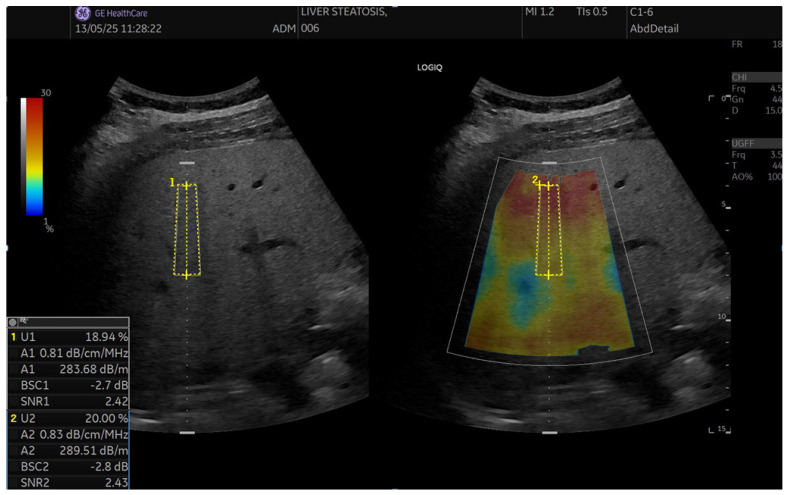
Example of UGFF acquisition. A homogeneous ROI (yellow) is placed in the right hepatic lobe ≥2 cm below the capsule, avoiding vessels. The system reports UGFF (%), attenuation coefficient (AC, dB/m or dB/cm/MHz), integrated backscatter coefficient (BSC, dB), and signal-to-noise ratio (SNR); the per-subject result was the median of 10 valid measurements per breathing condition.

SNR is another novel QUS parameter. It quantifies the homogeneity of B-mode image texture. SNR is known as a parameter of the Rayleigh distribution and is defined as the reciprocal of the Rayleigh parameter. In a normal healthy liver, the texture of B-mode images is not homogeneous due to the presence of structures such as blood vessels. On the contrary, in severe fatty liver, fat droplets become the dominant scatterers and mask these structures, resulting in a more homogenous texture. Consequently, SNR increases with the amount of fat droplets and the homogeneity of B-mode image texture. SNR is the ratio of the average and the standard deviation of the linear amplitude. The linear amplitude is calculated using tissue-harmonic B-mode data acquired under fixed transmission and reception conditions—Figure 1 and Figure 2.

Within each breathing condition, per-subject summaries were calculated as the median of the 10 valid acquisitions; these medians were used for primary analyses, and paired condition medians (apnea vs. free breathing) were used to assess breathing-related differences. Data quality was monitored using the system’s internal indicators (including SNR thresholds and motion flags); acquisitions with suboptimal quality were repeated, and datasets that failed to reach the predefined number of valid measurements were excluded. The protocol adhered to EFSUMB recommendations for liver elastography and was harmonized with prior GE-based QUS studies, including patient positioning, right-lobe intercostal access, ROI depth below the capsule, breath-hold acquisition, and standardized reliability and reporting conventions.

### 2.6. CAP Protocol

CAP was measured using a FibroScan^®^ Compact 530 (Echosens, Paris, France) during the same session as QUS. Examinations adhered to EFSUMB recommendations for transient elastography: supine position with the right arm maximally abducted, a right-lobe intercostal approach, and minimal probe pressure. The A-mode guide was used to confirm a homogeneous parenchymal window free of large vessels and bile ducts [16].

Probe selection (M or XL) followed manufacturer criteria based on body habitus/skin-to-capsule distance and the automatic probe-selection tool.

For each participant, 10 acquisitions were obtained in the right lobe; measurements rejected by the device were reacquired, and the median CAP (dB/m) was retained for analysis.

CAP reliability was predefined as CAP-IQR ≤ 40 dB/m, and a sensitivity analysis additionally applied the CAP-IQR/median < 0.30 threshold, consistent with prior clinical studies. Although WFUMB recommends a more stringent IQR/median < 15% criterion for attenuation-based measurements, the 30% threshold was selected in our study because it reflects the conventional reliability standards used in large CAP-validation cohorts and ensures methodological comparability with previous real-world clinical research. Steatosis grades were defined using CAP, with commonly reported cutoff values based on published studies and meta-analyses: S1 ≥ 230 dB/m, S2 ≥ 275 dB/m, and S3 ≥ 300 dB/m. These cutoff values served as comparators for QUS performance [8,10,17].

### 2.7. Outcomes

Primary outcomes were (i) linear correlation between each QUS parameter (UGFF, AC, BSC, SNR) and CAP; and (ii) diagnostic performance of each QUS parameter for detecting CAP-defined steatosis thresholds (≥S1, ≥S2, S3), expressed as area under the receiver operating characteristic (AUC) curve, sensitivity, specificity, positive predictive value (PPV), and negative predictive value (NPV). Sensitivity, specificity, and diagnostic accuracy were calculated using CAP as the reference standard, acknowledging that CAP is not a true gold standard.

Secondary outcomes included feasibility (rate of reliable acquisitions) and agreement between apnea and free-breathing QUS measurements assessed by Bland–Altman analysis (bias and limits of agreement).

### 2.8. Statistical Analysis

Statistical analyses were performed in MedCalc (MedCalc Version 19.4, MedCalc Software Ltd., Ostend, Belgium). Continuous variables are summarized as mean ± standard deviation for approximately normally distributed data or median (interquartile range) otherwise; normality was assessed with the Kolmogorov–Smirnov test. Categorical variables are presented as counts and percentages. For the breathing-maneuver comparison, only participants with paired breath-hold and free-breathing acquisitions were included; paired *t*-tests or Wilcoxon signed-rank tests were applied as appropriate. Within-condition measurement variability was summarized using the coefficient of variation (CV). Agreement between breathing conditions was assessed with Bland–Altman analysis (mean bias and 95% limits of agreement). Correlations between QUS metrics and CAP were evaluated with Pearson’s correlation coefficient (r) and 95% confidence intervals (via Fisher z-transformation). Diagnostic performance for CAP-defined steatosis thresholds (≥S1: 230 dB/m; ≥S2: 275 dB/m; S3: 300 dB/m) was assessed using receiver operating characteristic (ROC) analysis, reporting the area under the curve (AUC) with 95% CIs. Optimal cutoffs were determined by the Youden index; sensitivity, specificity, positive predictive value (PPV), and negative predictive value (NPV) are reported with 95% CIs. Pairwise AUC comparisons were performed using DeLong’s method. All analyses used available valid data without imputation. Two-sided *p* values < 0.05 were considered statistically significant.

## 3. Results

### 3.1. Baseline Characteristics

The study cohort included 196 patients, of whom 80 (40.8%) were women and 116 (59.2%) men, with a mean age of 52.4 ± 13.6 years (median 53, range 22–78). The mean BMI was 27.17 ± 7.52 kg/m^2^ (median 27.85, range 18–45). The mean CAP value was 250.16 ± 62.84 dB/m (median 242, range 142–400). To discriminate between steatosis stages by CAP, we used the cutoffs recommended by the manufacturer: S1 (mild)—230 dB/m; S2 (moderate)—275 dB/m; and S3 (severe)—300 dB/m [17]. Steatosis distribution according to CAP values, the baseline characteristics, demographic data, and laboratory parameters of the included patients are summarized in Table 1.

**Figure 3:**
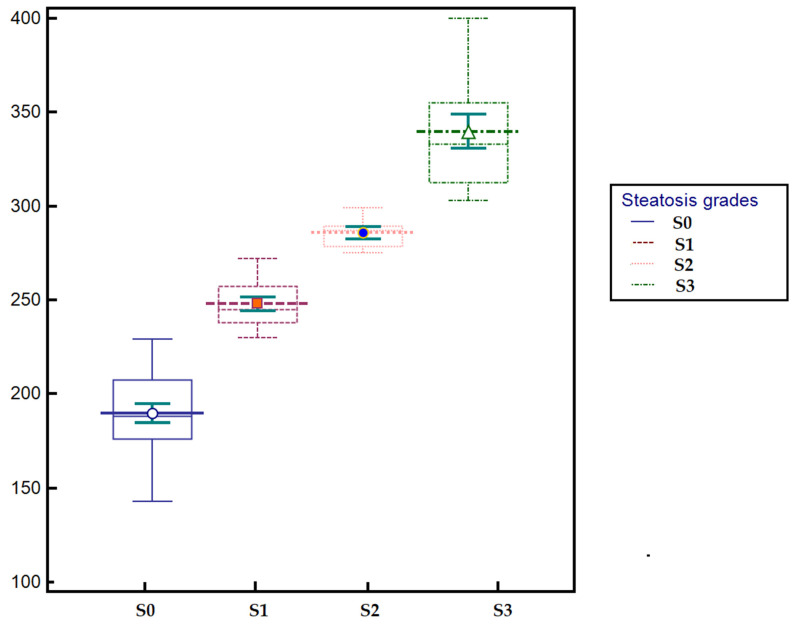
Box-plot illustrating the distribution of CAP values across steatosis grades (S0–S3). CAP values show a progressive increase with advancing steatosis severity, with the highest medians observed in patients with severe steatosis (S3).

Stratification by steatosis grade showed that patients with no steatosis (S0) had a mean CAP of 189.7 ± 22.45 dB/m (median 188 (143–229)), those with mild steatosis (S1) had 247.9 ± 12.5 dB/m (median 245, (230–272)), while patients with moderate steatosis (S2) had 285.9 ± 7.75 dB/m (median 287, (275–289)). The highest values were observed in patients with severe steatosis (S3), with a mean of 339.8 ± 30.43 dB/m (median 333, (303–400)). The distribution of CAP values across steatosis grades is illustrated in Figure 3.

**Figure 4:**
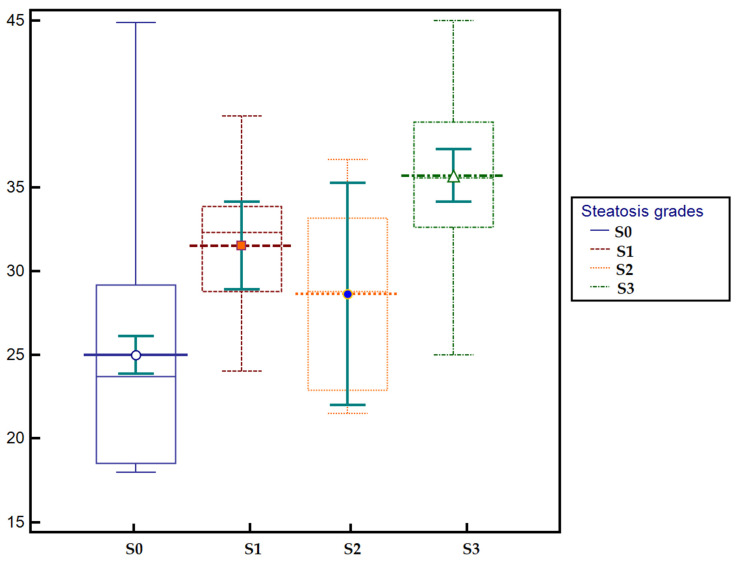
Box-plot illustrating the distribution of BMI values across steatosis grades (S0–S3). A gradual increase in BMI is observed from S0 (no steatosis) to S3 (severe steatosis), with higher medians in patients with advanced steatosis.

The mean BMI of the study cohort was 27.17 ± 7.52 kg/m^2^, with a median of 27.85 (range 18.0–45.0). According to steatosis grade, patients with S0 (no steatosis) had a mean BMI of 24.99 ± 6.36 (median 23.7, range 18.0–44.9), those with S1 (mild steatosis) had 31.53 ± 5.26 (median 32.35, range 20.5–41.8), while patients with S2 (moderate steatosis) had 28.65 ± 6.33 (median 28.8, range 21.5–36.7). The highest values were observed in S3 (severe steatosis), with a mean BMI of 35.73 ± 5.25 (median 35.6, range 25.0–45.0). The distribution of BMI values across steatosis grades is illustrated in Figure 4.

### 3.2. Effect of Breathing Conditions on the Reliability of Ultrasound Parameters

Reliable ultrasound measurements for all the evaluated parameters were successfully obtained in all 196 patients (100%) during apnea acquisitions. No cases failed due to limited intercostal spaces, high BMI, or suboptimal visualization. This unusually high feasibility may be related to the penetration capabilities of the LOGIQ E10 platform and the standardized right-intercostal acquisition protocol used in all examinations. In a subset of 126 patients (64.3%), paired acquisitions were also performed during free breathing; among these, valid measurements could not be obtained in only three cases (2.4%), confirming the high feasibility of both protocols. Comparative analysis of these patients showed no significant differences between apnea and free-breathing acquisitions for UGFF, BSC, and SNR values (all *p* > 0.05). For AC, the mean value was slightly higher during free breathing (231.6 ± 47.6) compared to apnea (225.9 ± 45.6) (*p* = 0.036). Although reliable measurements were less frequently obtainable during respiration, once valid acquisitions were achieved, the variability of the measurements (expressed as coefficient of variation) was similar between apnea and respiration across all parameters (Table 2).

**Figure 5:**
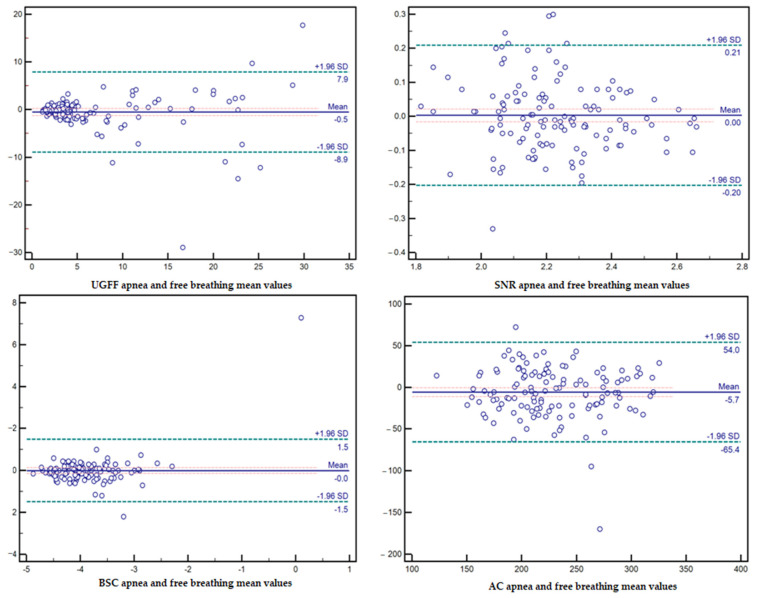
Bland–Altman plots comparing apnea and free-breathing acquisitions for all parameters. UGFF: mean bias −0.5, with 95% limits of agreement between −8.9 and 7.9. SNR: mean bias 0.0, with limits of agreement −0.20 to 0.21. BSC: mean bias −0.0, with limits of agreement −1.5 to 1.5. AC: mean bias −5.7, with limits of agreement −65.4 to 54.0.

Bland–Altman analysis confirmed good agreement between apnea and free-breathing acquisitions across all parameters. For UGFF, the mean bias was −0.5 (95% limits of agreement: −8.9 to 7.9), indicating minimal systematic difference. AC showed a small negative bias of −5.7 (95% LoA: −65.4 to 54.0), consistent with the slight shift observed in the paired analysis. For BSC, the bias was negligible (−0.0; 95% LoA: −1.5 to 1.5), and for SNR, the mean bias was virtually zero (0.0; 95% LoA: −0.20 to 0.21). Overall, these findings indicate that respiration does not substantially alter measurement reliability once valid acquisitions are obtained—Figure 5.

### 3.3. Predictive Performance of Ultrasound-Derived Parameters for Hepatic Steatosis and Their Correlation with CAP

**Figure 6:**
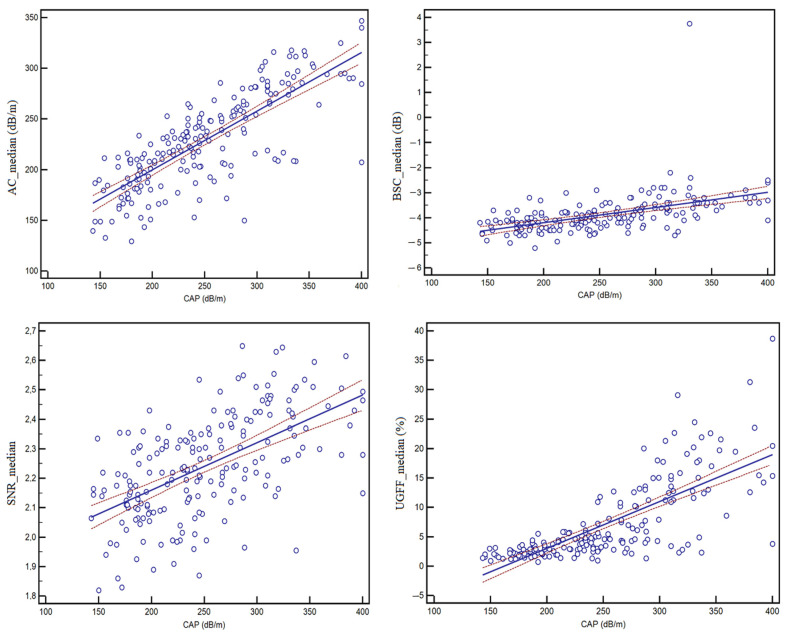
Scatter plots showing the correlation between CAP and each ultrasound-derived parameter: AC, BSC, SNR, and UGFF. Solid lines represent the linear regression fit with 95% confidence intervals (dashed lines). Strong correlations were found for AC (r = 0.79, 95% CI: 0.73–0.84) and UGFF (r = 0.75, 95% CI: 0.68–0.81), while BSC (r = 0.50, 95% CI: 0.38–0.60) and SNR (r = 0.58, 95% CI: 0.47–0.67) showed moderate correlations.

Correlation analysis showed a very strong association between AC and CAP (r = 0.79, 95% CI: 0.73–0.84, *p* < 0.0001), as well as between UGFF and CAP (r = 0.75, 95% CI: 0.68–0.81, *p* < 0.0001). Moderate correlations were observed for SNR (r = 0.58, 95% CI: 0.47–0.67, *p* < 0.0001) and BSC (r = 0.50, 95% CI: 0.38–0.60, *p* < 0.0001). Scatter plots with linear regression lines further illustrate the correlation between the quantitative ultrasound parameters and CAP, as depicted in Figure 6.

For all ROC and AUC analyses, the diagnostic performance of each QUS parameter was calculated using the apnea-based median values, as these acquisitions demonstrated the highest consistency and reliability in our cohort.

**Figure 7:**
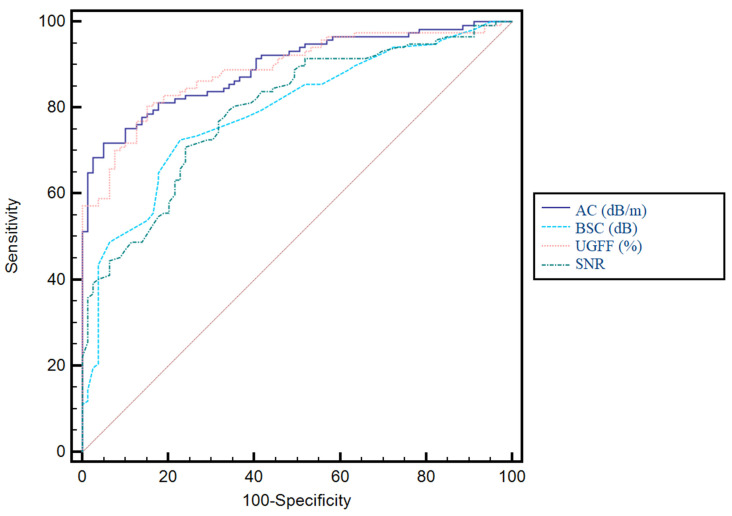
Receiver operating characteristic (ROC) curves for the assessment of any grade of steatosis. Comparison of AC (dB/m), BSC (dB), UGFF (%), and SNR. Both AC and UGFF demonstrated the highest diagnostic accuracy, whereas BSC and SNR showed lower performance.

For the prediction of any grade of steatosis, the best performing parameters were UGFF (%) and AC, both achieving an AUC of 0.89 (*p* < 0.0001), with no significant difference between them (*p* = 0.75). In contrast, AC showed significantly higher performance compared to BSC (*p* = 0.0007) and SNR (*p* = 0.0012). Similarly, UGFF outperformed both BSC (*p* = 0.0002) and SNR (*p* < 0.0001). No significant difference was observed between BSC and SNR (*p* = 0.89). These findings indicate that while all parameters demonstrated statistically significant diagnostic accuracy, AC and UGFF emerged as the most valuable predictors for detecting any grade of steatosis. The comparative diagnostic performance of the evaluated parameters is detailed in Table 3, while the corresponding ROC curves are illustrated in Figure 7.

**Figure 8:**
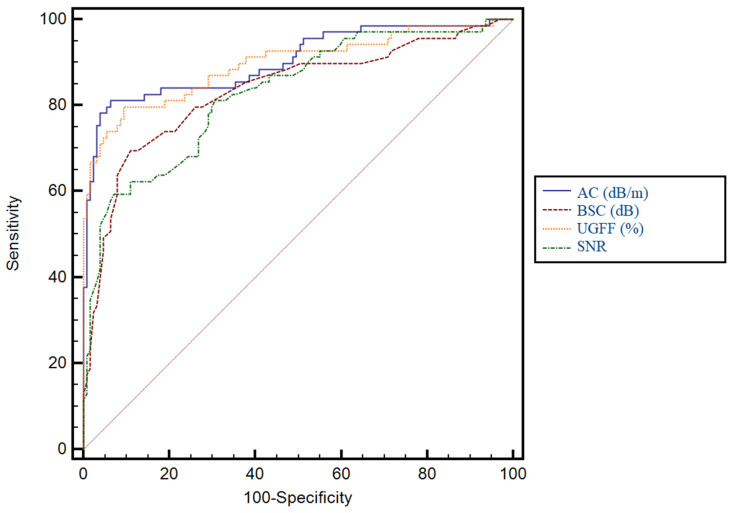
Receiver operating characteristic (ROC) curves for the assessment of significant steatosis. AC (dB/m) achieved the best performance, closely followed by UGFF (%), while BSC and SNR displayed inferior diagnostic accuracy.

For significant steatosis (moderate or severe steatosis), AC achieved the highest diagnostic accuracy with an AUC of 0.90 (*p* < 0.0001), being significantly superior to BSC (*p* = 0.0023) and SNR (*p* = 0.0048) but not different from UGFF (*p* = 0.53). UGFF also demonstrated excellent performance (AUC = 0.89), significantly outperforming both BSC (*p* = 0.001) and SNR (*p* = 0.001). No significant difference was noted between BSC and SNR (*p* = 0.85). Therefore, AC and UGFF represent the most robust parameters for detecting significant steatosis, whereas BSC and SNR showed lower diagnostic accuracy. Full details of the cutoff values, sensitivity, specificity, and predictive values for each parameter are provided in Table 4, while the corresponding ROC curves are illustrated in Figure 8.

**Figure 9:**
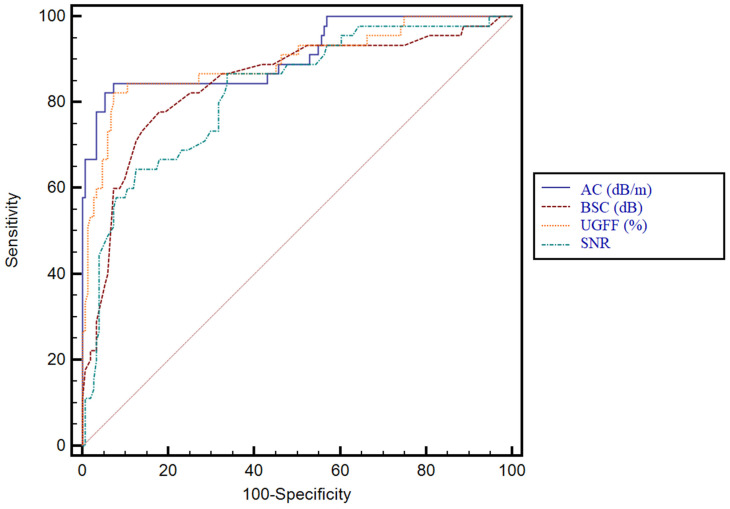
Receiver operating characteristic (ROC) curves for the assessment of severe steatosis. AC (dB/m) achieved the best diagnostic performance, closely followed by UGFF (%), while BSC and SNR showed lower accuracy.

For severe steatosis, AC achieved the highest diagnostic accuracy with an AUC of 0.91 (*p* < 0.0001), being significantly superior to BSC (*p* = 0.0033) and SNR (*p* = 0.0033) but not different from UGFF (*p* = 0.17). UGFF also demonstrated excellent performance (AUC = 0.89), significantly outperforming both BSC (*p* = 0.0106) and SNR (*p* = 0.0035). No significant difference was noted between BSC and SNR (*p* = 0.53). Therefore, AC and UGFF represent the most robust parameters for detecting severe steatosis, whereas BSC and SNR showed lower diagnostic accuracy. Full details of the cutoff values, sensitivity, specificity, and predictive values for each parameter are provided in Table 5, while the corresponding ROC curves are illustrated in Figure 9.

## 4. Discussion

Metabolic dysfunction–associated steatotic liver disease (MASLD, formerly NAFLD) is a growing global health concern, now affecting about 30%of adults and expected to impact over half by 2040 [1]. MASLD has become a leading cause of chronic liver disease and liver transplantation, particularly in Western nations. Most patients face increased cardiovascular risk, highlighting the need for early detection and intervention. Accurate, non-invasive quantification of liver fat is essential, as even mild steatosis poses metabolic and renal risks. The updated MASLD terminology stresses the importance of addressing metabolic dysfunction through targeted management and reliable point-of-care diagnostic tools [6,18].

After years of no approved pharmacotherapy for steatohepatitis, the therapeutic landscape is evolving. Resmetirom, a thyroid hormone receptor-β agonist, became the first FDA-approved medication targeting MASLD with significant fibrosis in 2024 in the USA. In the EU, a conditional marketing authorization was granted in August 2025, with launch starting in select countries. Clinical trials showed liver-fat reduction (MRI-PDFF) and histological benefits in MASH [19]. Other agents, like lanifibran, are in advanced Phase III trials for metabolic steatohepatitis. A recent Phase II study showed a 44% relative reduction in hepatic fat within 24 weeks, highlighting the achievable therapeutic endpoint of marked steatosis regression [20]. These developments highlight the urgent need for accessible and reproducible methods to quantify steatosis in trials and routine practice. Liver biopsy is invasive and impractical for serial monitoring in large populations; MRI-PDFF is accurate but less accessible and more costly. Ultrasound-based quantification techniques are widely accessible, radiation-free, and cost-effective, filling this gap. As new drugs enter clinical practice, clinicians will need practical tools to monitor treatment response in real time. QUS methods are gaining interest as a way to bring fat quantification to the point-of-care method, enabling longitudinal tracking of steatosis in large MASLD populations.

The CAP obtained via FibroScan has been a pioneering non-invasive technique for grading steatosis. In the past decade, CAP has been extensively validated against liver biopsy or MRI-PDFF [21,22]. Meta-analyses in biopsy-proven NAFLD show that CAP can detect histological steatosis with high sensitivity and acceptable specificity; for example, pooled estimates report AUROCs around 0.82–0.96 for diagnosing mild steatosis (≥S1) [23]. CAP’s strongest performance is ruling in or ruling out moderate-to-severe fatty change, making it a useful clinical surrogate for liver fat burden. In our study, we utilized CAP as the reference standard for steatosis grading owing to its wide availability and prior validation against both biopsy and MRI-PDFF. This pragmatic choice reflects real-world practice, where CAP is often the most accessible quantitative assessment tool for liver fat in clinics.

At the same time, several practical constraints of CAP help explain the interest in imaging-guided alternatives. CAP is a dedicated, single-purpose device that reports only an attenuation metric (dB/m) and does not provide concurrent morphological assessment, Doppler, or contrast-enhanced imaging. By contrast, modern ultrasound devices increasingly integrate steatosis quantification alongside routine abdominal ultrasound in the same session. It is also important to acknowledge that CAP-based categories (S1–S3) are pragmatic clinical thresholds rather than biological ground truth, as true steatosis severity is defined by histological fat percentage.

Recent years have seen rapid development of QUS techniques, which directly measure tissue acoustic properties, moving beyond qualitative B-mode echogenicity.

The AC quantifies how much acoustic energy is lost as the beam traverses liver parenchyma; greater intrahepatic fat increases attenuation, making AC a direct, quantitative surrogate for steatosis. Building on this principle, vendors have implemented AC-based tools with strong MRI-PDFF concordance and high diagnostic accuracy. On GE HealthCare systems (UGAP, LOGIQ platform), a large multicenter cohort reported AUROCs ≈ 0.89–0.91 across MRI-PDFF–defined steatosis grades and good agreement with MRI-PDFF, supporting clinical use for grading [24]. Samsung Medison’s TAI (often paired with TSI) similarly shows robust performance, with prospective studies demonstrating AUC ≈ 0.89–0.93 for MRI-PDFF thresholds of ≥5% and ≥10%, alongside strong correlation and good reproducibility [25]. Canon’s ATI (Aplio i800) correlates strongly with MRI-PDFF (r ≈ 0.81) and yields AUROCs of 0.91 (≥5%) and 0.95 (≥16.3%) [26]. Fujifilm’s iATT also shows high accuracy in a multicenter study, with significant correlation to MRI-PDFF (r = 0.73) and ROC analyses supporting reliable discrimination across steatosis grades [27]. Other ultrasound systems also incorporate such QUS.

BSC quantifies the fraction of acoustic energy returned to the transducer after interaction with liver microstructure; fat infiltration alters acoustic impedance and micro-scatterers, increasing backscatter relative to normal parenchyma [28]. Building on this principle, Tissue Scatter Distribution Imaging (TSI)—available on Samsung Medison systems (e.g., RS85/Prestige)—analyzes the statistical distribution of the backscattered signal within a defined ROI; across six prospective cohorts, TSI has shown excellent pooled AUC (~0.91) with moderate correlation to MRI-PDFF for steatosis detection, supporting clinical feasibility [25].

Recently, ultrasound manufacturers have developed and implemented ultrasound fat fraction techniques that combine several parameters, such as attenuation and BSC. Ultrasound fat-fraction estimation tools developed by different vendors have shown strong agreement with MRI-PDFF and clearly outperform conventional B-mode. On Siemens Healthineers systems, Labyed and Milkowski (2020) published the first experimental on-system UDFF study using an integrated reference phantom; the algorithm combines AC and BSC to estimate liver fat and showed strong agreement with MRI-PDFF (r = 0.87), with AUCs of 0.97 and 0.95 for MRI-PDFF ≥5% and ≥10%, respectively [29]. De Robertis et al. prospectively evaluated 122 patients, showing a strong correlation with MRI-PDFF (ρ = 0.81) and superior performance to conventional B-mode for steatosis detection (AUC 0.75 vs. 0.53) [30]. More recently, a 2025 prospective study by Jin et al. reported a UDFF AUC of 0.981 (vs. 0.932 for CAP) for MRI-PDFF ≥5%, with a correlation of r = 0.876 with MRI-PDFF [31]; a 2025 meta-analysis pooling eight studies showed a pooled Se of 90.4%/Sp 83.8%, with an AUC of 0.93 for the summary receiver operating characteristic curve for UDFF [32]. Recent evidence summarized by Kavvadas et al. (2025) indicates that Siemens UDFF achieved excellent correlation with MRI-PDFF (r ≈ 0.85) and high diagnostic accuracy (AUC ≥ 0.89)for steatosis grading [33]. On Samsung Medison platforms, Kaposi et al. (2023) proposed a multivariable ultrasound-estimated fat fraction (USFF) model combining Tissue Attenuation Imaging (TAI™) and Tissue Scatter Distribution Imaging (TSI™) to derive attenuation and backscatter-based parameters [15]. In their cohort, the USFF showed very strong correlation with MRI-PDFF (r = 0.83) and excellent diagnostic performance for ≥5% and ≥10% steatosis (AUCs up to 0.99 and 0.96, respectively) [15]. Yin et al. (2024) showed that quantitative ultrasound system fat fraction (USFF, Samsung Medison) correlates well with proton magnetic resonance spectroscopy (r = 0.76) and delivers high diagnostic performance (AUC 0.84–0.98), outperforming CAP and US grading for steatosis assessment [34].

Moreover, when three parameters were included, in a multicenter prospective study, Kuroda et al. (Radiology, 2023) showed that a multivariable QUS model combining UGAP (attenuation), IBSC (backscatter), and SNR accurately discriminated ≥5% steatosis versus MRI-PDFF, with AUCs up to 0.96—supporting the rationale for our composite UGFF parameter, which integrates attenuation, backscatter, and SNR into a single fat-fraction estimate [35].

Our results support these trends. In the present study, the novel ultrasound-guided fat fraction (UGFF) showed excellent discrimination for significant steatosis, with an AUC of 0.89 (95% CI: 0.84–0.93; *p* < 0.0001) at a 6% cutoff, yielding 79.7% sensitivity, 90.5% specificity, a PPV 82.1% of, and an NPV of 89.1%.This performance is in line with contemporary QUS fat-fraction literature: UDFF/USFF typically correlates strongly with MRI-PDFF and delivers high diagnostic accuracy (e.g., De Robertis et al. reported ρ ≈ 0.81 with an AUC of 0.75 vs. B-mode 0.53; Yin et al. found USFF AUCs of ~0.84–0.98 and superiority over CAP; a 2025 meta-analysis pooled Se 90.4%/Sp 83.8% with sROC-AUC 0.93; and a 2025 head-to-head study showed UDFF AUC 0.981 vs. CAP 0.932 for MRI-PDFF ≥ 5%) [30,34]. Notably, absolute cutoffs vary across vendors and by reference standard (CAP vs. MRI-PDFF), so the optimal 6% threshold here should be interpreted as device- and reference-specific rather than directly transferable to other platforms or MRI-based criteria.

The strong correlation we observed between UGFF and CAP (r ≈ 0.75) is also consistent with previous head-to-head comparisons. In a mixed-etiology liver disease cohort, Sporea et al. reported a good correlation between UDFF and CAP and an increased UDFF specificity in severe steatosis cases. Notably, that study reported UDFF had increased specificity in severe steatosis, suggesting better discrimination at high fat levels. Other groups have gone further to compare UDFF against CAP and serum indices: in high-MASLD-risk patients, Tavaglione et al. showed that UDFF achieved an AUC of 0.89 (vs. CAP-defined steatosis) and outperformed traditional biomarkers, such as the fatty liver index (FLI) and hepatic steatosis index [36]. Together, these data position multiparametric QUS—particularly attenuation–backscatter combinations—as a true quantitative alternative to CAP, with diagnostic efficacy approaching that of MRI-PDFF. In practical terms, this means an ultrasound exam could potentially provide an instant liver fat percentage that correlates strongly with MRI-PDFF and even histology, enabling clinicians to diagnose and grade MASLD non-invasively with confidence.

This study also examined how breathing maneuvers affect measurement values and variability. In the paired subset with valid measurements in both conditions (*n* = 126), UGFF, BSC, and SNR showed no significant differences between apnea and free-breathing acquisitions (all *p* > 0.05), whereas AC was slightly higher during free breathing (231.6 ± 47.6 vs. 225.9 ± 45.6 dB/m; *p* = 0.036). The slightly higher AC values observed during free breathing may be explained by mild respiratory motion, which introduces frame-to-frame decorrelation and subtle blurring of the ultrasound beam path. These effects can influence the depth-dependent spectral decay used in attenuation estimation, leading to a small apparent increase in AC during respiration. Although valid datasets were less frequently obtainable during free breathing, once valid frames were acquired, the within-condition variability (coefficient of variation) was comparable across respiration states for all parameters. These observations are consistent with contemporary evidence that attenuation- and backscatter-based techniques are largely robust to respiratory motion once quality criteria are met: in pediatric cohorts, ATI and UGAP medians do not differ between free-breathing and breath-hold and show excellent reproducibility, with only slightly higher variability in younger children during free-breathing [37]; UDFF studies across multiple breathing/postural conditions likewise report no systematic mean shifts and the lowest coefficients of variation during supine breath-hold, indicating that apnea chiefly improves precision rather than correcting bias [38]. Methodological standardization papers for AC further demonstrate the highest repeatability with brief breath-hold, right-lobe intercostal access, and an ROI placed ≥2 cm below the capsule—principles adopted in our protocol [39]. Finally, feasibility data in children suggest that stable free-breathing can be acceptable when cooperation with breath-hold is limited, with maintained diagnostic performance under standardized acquisition criteria [40]. Collectively, these data support using a simple breath-hold whenever feasible to maximize feasibility and precision, while accepting free-breathing acquisitions in non-cooperative patients provided predefined quality thresholds are satisfied.

This study has several limitations. First, we used CAP as our reference standard, which—like AC—measures ultrasonic attenuation; thus, observed concordance may reflect methodological similarity rather than true diagnostic accuracy. Therefore, the diagnostic performance reported in this study should be interpreted as agreement with CAP, not as absolute accuracy versus histology. Moreover, the reliance on CAP as a reference standard—given its shared attenuation-based ultrasound methodology—introduces an inherent methodological dependency; therefore, definitive assessment of UGFF performance requires external validation against independent standards such as MRI-PDFF or histological quantification. Our results should be considered in concordance with CAP, not definitive accuracy, and future research should compare QUS metrics to independent standards such as MRI-PDFF or histology. Second, the single-center design with all measurements performed on one machine enhances internal validity but limits generalizability; performance may vary with different ultrasound systems or clinical settings. Standardization of calibration and cutoff values is essential as QUS adoption expands. Third, selection bias may exist, as the cohort primarily included patients referred for liver evaluation, resulting in a high prevalence of steatosis.

Despite these limitations, our findings carry several practical implications. The strong diagnostic performance of UGFF, coupled with its non-invasiveness, supports the idea that UGFF can be implemented for MASLD management. For clinicians, this means a standard abdominal ultrasound exam could generate quantitative steatosis data (e.g., “liver fat = 10%”) in addition to routine fibrosis assessment by ultrasound-based elastography. Such a tool could be used to screen and risk-stratify patients (identifying those with clinically significant steatosis who might benefit from lifestyle or pharmacologic interventions), as well as to monitor response to therapy in a way that is far more accessible than serial MRI. The use of CAP in clinical practice has already shown the demand for point-of-care steatosis assessment; our results indicate that newer QUS methods can match, if not improve upon, CAP’s performance while providing a more direct fat quantification. Looking ahead, multiparametric QUS is poised to become a cornerstone of MASLD evaluation, analogous to how elastographic techniques are now standard for fibrosis. In summary, the present study adds to the growing literature affirming that QUS-based hepatic fat assessment is accurate, reproducible, and clinically meaningful, offering a promising solution to the urgent need for scalable MASLD evaluation tools in the era of emerging therapies.

## 5. Conclusions

UGFF shows strong agreement with CAP across steatosis grades and similar within-subject variability during breath-hold and free-breathing once valid measurements are obtained. These results support UGFF as a practical tool for liver fat assessment. Further confirmation against MRI-PDFF or histology and multicenter studies is needed.

## Figures and Tables

**Table 1:** Characteristics of the included subjects.

Parameter	Subjects *n* = 196
Mean age (years)	52.40 ± 13.6
Gender	
Males	116/196 (59.2%)
Females	80/196 (40.8%)
Mean BMI (kg/m^2^)	27.17 ± 7.52
Abdominal circumference (cm)	94.60 ± 11.04
AST (U/L)	49.19 ± 10.80
ALT(U/L)	38.17 ± 13.60
GGT (U/L)	93.41 ± 32.57
Cholesterol (mg/dL)	192.11 ± 33.43
Triglyceride (mg/dL)	148.69 ± 34.19
Platelet count (×10^9^/L)	242.40 ± 57.32
Total bilirubin (mg/dL)	0.82 ± 0.34
Steatosis distribution	
S0	79 (40.3%)
S1	48 (24.5%)
S2	24 (12.2%)
S3	45 (23%)

Values are expressed as mean ± standard deviation (SD) unless otherwise indicated. Steatosis distribution is reported according to Controlled Attenuation Parameter (CAP)–derived grades: S0—no steatosis, S1—mild steatosis, S2—moderate steatosis, S3—severe steatosis. BMI—body mass index, AST—aspartate aminotransferase, ALT—alanine aminotransferase, GGT—gamma-glutamyl transferase.

**Table 2:** Comparison of ultrasound-derived parameters between apnea and free-breathing acquisitions.

Parameter	Apnea Acquisition (Mean ± SD)	Free-Breathing Acquisition (Mean ± SD)	CV Apnea (%)	CV Free Breathing (%)	*p*-Values
UGFF (%)	6.71 ± 7.10	7.23 ± 7.18	105.8	99.3	0.177
AC (dB/m)	225.86 ± 45.64	231.59 ± 47.60	20.2	20.6	0.036
BSC (dB)	−3.91 ± 0.85	−3.90 ± 0.52	21.9	13.2	0.953
SNR	2.23 ± 0.18	2.22 ± 0.19	7.9	8.5	0.754

Values are expressed as mean ± standard deviation (SD). Variability is reported using the coefficient of variation (CV). Paired comparisons were performed using paired *t*-test and Wilcoxon signed-rank test. UGFF—ultrasound-guided fat fraction, AC—attenuation coefficient, BSC—backscatter coefficient, SNR—signal-to-noise ratio, CV—coefficient of variation.

**Table 3:** Diagnostic performance of ultrasound-derived parameters for predicting any degree of steatosis (≥S1).

Parameter	AUC	95% CI	*p*-Value	Cutoff Value	Se	Sp	PPV	NPV
UGFF (%)	0.89	0.83–0.92	<0.0001	4%	77%	84.8%	88.2%	71.3%
AC (dB/m)	0.89	0.84–0.93	<0.0001	232	71.8	94.9	95.5	69.4
BSC (dB)	0.78	0.72–0.84	<0.0001	−4	72.6	77.2	82.5	65.6
SNR	0.79	0.73–0.84	<0.0001	2.2	70.9	75.9	81.4	63.8

Values are expressed as the area under the receiver operating characteristic curve (AUC) with a 95% confidence interval (CI). Cutoff values were selected according to the Youden index. Sensitivity (Se), specificity (Sp), positive predictive value (PPV), and negative predictive value (NPV) are reported.

**Table 4:** Diagnostic performance of ultrasound-derived parameters for predicting significant steatosis (≥S2).

Parameter	AUC	95% CI	*p*-Value	Cutoff Value	Se	Sp	PPV	NPV
UGFF (%)	0.89	0.84–0.93	<0.0001	6%	79.7%	90.5%	82.1%	89.1%
AC (dB/m)	0.90	0.85–0.94	<0.0001	250	81.2	93.7	87.5	90.2
BSC (dB)	0.83	0.77–0.88	<0.0001	−3.75	69.6	89	77.4	84.3
SNR	0.82	0.76–0.87	<0.0001	2.4	59.4	92.9	82	80.8

Values are expressed as area under the receiver operating characteristic (AUC) curve with 95% confidence interval (CI). Cutoff values were selected according to the Youden index. Sensitivity (Se), specificity (Sp), positive predictive value (PPV), and negative predictive value (NPV) are reported.

**Table 5:** Diagnostic performance of ultrasound-derived parameters for predicting severe steatosis (S3).

Parameter	AUC	95% CI	*p*-Value	Cutoff Value	Se	Sp	PPV	NPV
UGFF (%)	0.89	0.84–0.93	<0.0001	11%	80%	92.7%	77.6%	94.1%
AC (dB/m)	0.91	0.86–0.94	<0.0001	262	84.4	92.7	77.6	95.2
BSC (dB)	0.84	0.78–0.89	<0.0001	−3.7	77.8	82.1	56.5	92.5
SNR	0.82	0.76–0.87	<0.0001	2.45	57.8	92	68.4	88

Values are expressed as area under the receiver operating characteristic (AUC) curve with 95% confidence interval (CI). Cutoff values were selected according to the Youden index. Sensitivity (Se), specificity (Sp), positive predictive value (PPV), and negative predictive value (NPV) are reported.

## Data Availability

Data are available upon request by contacting the corresponding author at the following email address: bende.renata@umft.ro, due to privacy and ethical restrictions related to patient confidentiality.

## Abbreviations

The following abbreviations are used in this manuscript:

AC	Attenuation Coefficient
ALD	Alcoholic Liver Disease
ALT	Alanine Aminotransferase
AST	Aspartate Aminotransferase
AUC	Area Under the (ROC) Curve
AUROC	Area Under the Receiver Operating Characteristic Curve
B-mode	Brightness-Mode Ultrasound
BA	Bland–Altman
BMI	Body Mass Index
BSC	Backscatter Coefficient
CAP	Controlled Attenuation Parameter
CAP-IQR	Interquartile Range of CAP measurements
CI	Confidence Interval
CT	Computed Tomography
CV	Coefficient of Variation
dB/m	Decibels per meter
EFSUMB	European Federation of Societies for Ultrasound in Medicine and Biology
GGT	Gamma-Glutamyl Transferase
HBV	Hepatitis B Virus
HCV	Hepatitis C Virus
HDV	Hepatitis D Virus
IQR	Interquartile Range
LoA	Limits of Agreement
MAFLD	Metabolically Associated Fatty Liver Disease
MASLD	Metabolic Dysfunction-Associated Steatotic Liver Disease
MASH	Metabolic Dysfunction-Associated Steatohepatitis
MRI	Magnetic Resonance Imaging
MRI-PDFF	MRI–Proton Density Fat Fraction
NAFLD	Non-Alcoholic Fatty Liver Disease
NASH	Non-Alcoholic Steatohepatitis
NPV	Negative Predictive Value
PPV	Positive Predictive Value
QIBA	Quantitative Imaging Biomarkers Alliance
QUS	Quantitative Ultrasound
ROC	Receiver Operating Characteristic
ROI	Region of Interest
SD	Standard Deviation
SNR	Signal-to-Noise Ratio
S0–S3	CAP-Defined Steatosis Grades (none/mild/moderate/severe)
T2DM	Type 2 Diabetes Mellitus
TGC	Time-Gain Compensation
UGFF	Ultrasound-Guided Fat Fraction (ultrasound fat fraction)
